# Supplementary material for: Coordinated changes in gene expression, H1 variant distribution and genome 3D conformation in response to H1 depletion
Source: Nucleic Acids Res. 2022 Apr 5;50(7):3892–910. doi: 10.1093/nar/gkac226 (PMC9023279; doi:10.1093/nar/gkac226)

Coordinated changes in gene expression, H1 variant distribution and genome 3D conformation in response to H1 depletion

Núria Serna-Pujol[1,#], Mónica Salinas-Pena[1,#], Francesca Mugianesi[2,#], François Le Dily[3], Marc A. Marti-Renom[2,3,4,5], Albert Jordan[1,*]

SUPPLEMENTARY MATERIAL:
SUPPLEMENTARY TABLES
SUPPLEMENTARY FIGURE LEGENDS
SUPPLEMENTARY METHODS

Supplementary Table 1. Hi-C experimental statistics. Statistics shown separated by replicates (top table) and merged datasets (middle table for valid pairs and bottom table for filtered reads).

Supplementary Table 2. Mass-spectrometry analysis of histone H1 peptides after immunoprecipitation with H1 variant specific antibodies.

SUPPLEMENTARY FIGURE LEGENDS

Supplementary Figure 1. Characterization of multiH1 KD cells and ChIP-grade H1 antibodies. (A) The effect of H1 KD was confirmed by RT-qPCR, showing inhibition of multiple H1 variant genes and induction of satellites, repeats and interferon stimulated genes (ISGs). RNA extracted from T47D multiH1 KD cells cultured in the presence or not of Doxycycline for 6 days was reverse transcribed with random hexamers. Resulting cDNA was submitted to real-time PCR with oligos to measure expression of H1 variants genes, ISGs (IFI27 and OASL), satellites (SATa) and endogenous retroviruses (MER4D, MER21C and MLT1C49). Expression was represented relative to GAPDH as a control and relative to untreated cells. A representative experiment quantified in triplicate is shown. **(B)** Cell cycle profile after propidium iodide staining of mutliH1 KD cells growing for 6 days in the absence or presence of Dox. Data is expressed as percentage of cells in G1, S and G2/M phases. Values represent the mean and SD of six replicates. Statistical differences between -Dox and +Dox are supported by paired-t-test. (**) $P < 0.001$; (*) $P < 0.05$; (ns/non-significant) $P>0.05$. **(C)** H1 antibodies recognize its specific recombinant variant. Recombinant human histone H1 variants (125 ng) were immunoblotted with the indicated antibodies or Coomassie stained as a loading control. A total histones extract from T47D cells (10µg) was added as a control. All recombinant H1 variants are native except for 6xHis-HA-H1.2 and 6xHis-H1.4. **(D-H)** The specificity of H1 antibodies was further tested on Western blot and ChIP-qPCR with chromatin extracts from single H1

KD T47D cells, and with chromatin extracts from HeLa and HCT-116 cells, which lack H1.0 or H1.5, respectively. **(D-E)** Immunoblot analysis of H1 variants expression in inducible knock-down T47D cells for different H1 variants. Total histones (**D**) or chromatin (**E**) extracted from untreated or 6-days-Dox-treated single-H1 (H1.0 to H1.5, H1X) KDs cells were immunoblotted with the indicated antibodies or Coomassie stained as a loading control. T47D single-H1 KDs performance was previously characterized in Sancho et al., 2008 (ref.19 of manuscript). Immunoblot ImageJ quantification is shown in (**E**) and expressed relative to Untreated (-Dox). **(F)** ChIP-qPCR of H1 variants in inducible knock-down T47D cells for different H1 variants. Chromatin samples shown in (**E**) were used to perform ChIP with antibodies against H1 variants. Resulting DNA was amplified by qPCR with oligos for distal promoter (3kb upstream TSS) and TSS regions of genes CDK2 and NANOG. ChIP amplification is shown relative to input DNA amplification. A representative experiment is shown. Statistical differences between Untreated (-Dox) and +Dox immunoprecipitated DNA for each H1 variant are supported by paired-t-test. (***) $P < 0.001$; (**) $P < 0.01$; (*) $P < 0.05$; (ns/non-significant) $P>0.05$. **(G)** Immunoblot analysis of H1 variants expression within cell lines. Chromatin extracts (10 µg of protein) from T47D, HeLa and HCT-116 cells were run in SDS/PAGE and immunoblotted with the indicated antibodies against histone H1 variants or histone H3 as a loading control. **(H)** ChIP-qPCR of H1 variants in HeLa and HCT-116 cells. Chromatin was used for ChIP with antibodies against H1 variants and unrelated IgG as a control. Resulting DNA was amplified by qPCR with oligos for distal promoter (3kb upstream TSS) and TSS regions of genes CDK2 and NANOG. ChIP amplification is shown relative to input DNA amplification. A representative experiment is shown. **(I)** Only H1.4 was detected after H1.4 immunoprecipitation from histones extract. Total histones from T47D H1.4shRNA cells untreated or 6-days Dox-treated were immunoprecipitated with the H1.4 antibody. Immunoblot analysis with the indicated histone H1 antibodies was performed on input and immunoprecipitated material. Coomassie staining of samples is shown as a loading control.

Supplementary Figure 2. Genomic distribution of five histone H1 variants in human breast cancer cells. IGV browser screenshots of human chromosome 11 (partial) showing input-subtracted ChIP-seq abundance of H1 variants and H3K9me3 in T47D breast cancer cells under basal (-Dox) or multiple H1 depletion (+Dox). Increased chromatin accessibility upon H1 depletion is shown by differential ATAC-Seq signal (+/-Dox). TADs (TAD borders and TADs classified in four groups according to their H1.2/H1X ratio) and A/B compartments derived from Hi-C experiments under the same conditions are also shown. Under basal conditions, H1 variants are distributed in two large groups depending on the local GC content: H1X and H1.4 preferentially occupy active regions characterised by high-GC, A comparment and gene-richness (Region A in the zoom-in) while H1.0, H1.5 and H1.2 are enriched in low-GC chromatin, B compartment and gene-poor regions (Region B in the zoom-in). Upon H1 depletion, H1X increases its relative enrichment at high-GC regions while remaining H1.4 moves to the low-GC H1 group. Chromatin accessibility is specially increased in A comparment.

Supplementary Figure 3. Abundance of human histone H1 variants within Giemsa bands and chromatin states. (A) Box plots showing H1 variants and H3K9me3 input-subtracted ChIP-seq abundance within G bands, for each band type (Gneg1-4 and

Gpos25-100), in multiH1 KD cells treated or not with Doxycycline. GC content of G bands for each band type is also represented. Gneg bands were divided in 4 equal groups according to GC content. Wilcoxon signed-rank test was used to compare the H1 variants abundance within G bands before and after multiH1 KD: (***) $P < 0.001$; (**) $P < 0.01$; (*) $P < 0.05$. Gpos25, N = 87; Gpos50, N = 121; Gpos75, N = 89; Gpos100, N = 81; Gneg, N = 414. Data corresponds to a representative ChIP-seq experiment. **(B)** Scatter plots of the indicated H1 variant pairs input-subtracted ChIP-seq abundance within 100-kb bins of the human genome. The GC content at each bin is color-coded. Pearson's correlation coefficient is shown (*P*-value<0.001). **(C)** Scatter plot of H1.2 and H1X input-subtracted ChIP-seq abundance within Gpos and Gneg bands from T47D multiH1sh untreated cells. Pearson's correlation coefficient is shown (*P*-value<0.001). **(D)** Heat map and cluster analysis of the input-subtracted ChIP-seq abundance of H1 variants from WT or H1 KD T47D cells (-/+Dox) within a random sample of genome fragments belonging to the 10 chromatin states. For each chromatin state 1,000 fragments were randomly picked and 50 groups of 20 fragments were randomly generated. Each lane of the heat map represents the median input-subtracted ChIP-seq abundance of H1 variants in a group. Clustering confirmed that H1X was the most extreme variant and H1.4 showed an intermediate distribution together with H3K9me3.

Supplementary Figure 4. Functional characterization of the regions enriched for the different H1 variants in multiH1 KD T47D cells. (A) Average, input-subtracted ChIP-seq signal of H1 variants (-/+Dox) around TSS, TTS and gene bodies, grouped according to the basal expression of genes was calculated with CEAS (Cis-regulatory Element Annotation System, v0.9.9.7). 10% of total expressed genes in each group 1-10, being group 1 top expressed genes and group 10 genes with the lowest expression. Group 0 includes non-expressed genes according to RNA-seq data on T47D cells. Average for all genes is shown in black. Gene regions are represented as a 3-kb-long meta-gene surrounded by 1kb region upstream TSS and 1kb downstream TTS. In the upper panels, TSS or TTS are surrounded by 3kb regions. **(B)** Genomic annotation of regions enriched for the different H1 variants (-/+Dox). SICER (v1.1) was used to identify histone ChIP-enriched regions with the following parameters: redundancy threshold = 1, window size = 200, fragment size = 150, effective genome fraction = 0.80, gap size = 200 and FDR = 0.01. Genomic annotation of the identified H1-enriched regions was performed with CEAS software.

Supplementary Figure 5. Immunofluorescence of H1 variants in T47D breast cancer cells. (A) T47D wild-type cells were fixed and stained with the indicated antibodies. ChIP-grade anti-H1.2, H1.4, H1.5, H1X and H1.0 antibodies were used. DNA was labeled with DAPI Conjugated secondary antibodies anti-rabbit Alexa 488 and anti-mouse Alexa 555 were used. As both LaminA and H1.0 antibodies are raised in mouse, we used H1.4 to co-localize with H1.0, showing that they occupy different regions. Scale bar = 5μm. **(B)** T47D multiH1 KD cells cultured in the presence or not of Doxycycline for 6 days were fixed and stained with the corresponding primary antibodies used in (**A**). DNA was labeled with DAPI. Conjugated secondary Antibody anti-rabbit Alexa 647 or anti-mouse Alexa 633 were used. No co-localization experiments could be performed due to Untreated cells are RFP+ whereas Dox-treated cells express both RFP and GFP (for cell

line construction details see Reference 22 of the main manuscript). Scale Bar= 5μm. Bottom boxplot pannels show Immunofluorescence quantification under Untreated and Dox conditions, measured by Corrected Total Cell Fluorescence (see Supplementary methods). Number of cells used for quantification(-/+Dox): n=35 (H1X), n=30 (H1.4), n=55(H1.5), n=50 (H1.2), n=70(H1.0). Statistical differences are supported by paired-t-test: (ns=non-significant) P >0.05; (***) P < 0.001.

Supplementary Figure 6. Hi-C determination of genome interactions in H1 KD T47D cells. (A) Hi-C interaction maps for all 3 replicates and the merged maps of WT and H1 KD. Left plots correspond to WT maps and right plots are H1 KD maps. Rows show maps for replicates 1 to 3 as well as the merged maps. Each panel includes a Hi-C raw interaction maps at 1Mb resolution and 100 kb resolution for genome-wide and chromosome 1, respectively. **(B)** Plot comparing the distribution of Hi-C interactions versus genomic distance across the genome for a maximum distance of 500 Mb for WT and H1 KD cells for replicates 1 to 3. **(C)** Differential Hi-C map. Increased (red colored) and decreased (blue colored) interactions in contact matrices of chromosome 17 (0-20Mb) of H1 KD compared to WT cells, at 100kb bins resolution, for replicates 1 to 3. **(D)** Scatter plot of principal component (PC) coefficients for 100-kb genomic segments (bins) from WT (-Dox) and multiH1 KD (+Dox) cells for replicates 1 to 3. PC coefficients were used to define A (positive PC) and B (negative PC) compartments, as well as compartment shifting (A-to-B and B-to-A) upon H1 KD. **(E)** Box plot showing average number of differential intra- or inter-TAD interactions per chromosome upon H1 KD in different compartments, at 100 kb resolution, for replicates 1 to 3. The average number per chromosome of differential interactions for each category is indicated in the ticks of X axes.

Supplementary Figure 7. A/B compartments redistribution and H1 variants content within chromosomes. Scatter plot between the percentage of bins that changed from A to B or vice versa upon H1 KD, and the average H1 variants ChIP-seq signal in untreated cells within TADs, for each chromosome. The scatter plot between the percentage of bins in A or B compartment for each chromosome and the H1 variants ChIP-seq signal within TADs is also shown as a reference. Spearman's correlation coefficient is shown as well as *P*-value. Each point represents a different chromosome. Related to Figure 2D.

Supplementary Figure 8. Characterization of TADs containing genes up- or down-regulated upon multiH1 KD. (A) Number of TADs containing 0 or ≥1 up- or down-regulated genes (FC≥±1.4, adjusted p-value <0.05) upon H1 KD. 1,806 TADs contain no genes deregulated (including 70 TADs containing no genes); 1,292 TADs contain at least 1 deregulated gene: 531 TADs contain only up-regulated genes, 520 TADs contain only down-regulated genes, 241 TADs contain both up- and down-regulated genes. **(B)** Number of TADs containing 0, 1, 2, 3 or ≥4 up- or down-regulated genes (FC≥±2, adjusted p-value <0.05) upon multiH1 KD, measured by RNA-seq. 2,736 TADs contain no genes deregulated (including 70 TADs containing no genes); 362 TADs contain at least 1 deregulated gene: 176 TADs contain only up-regulated genes, 167 TADs contain only down-regulated genes, 19 TADs contain both up- and down-regulated genes. **(C)** Box

plot showing the gene expression change upon H1 KD (+/-Dox), H1.2 and H1X ChIP-seq signal in untreated cells, normalized gene richness and normalized basal gene expression within TAD groups: all TADs, TADs without deregulated genes upon H1 KD (Control), TADs containing only up-regulated genes, only down-regulated, or both up- and down-regulated genes simultaneously (FC≥±2, *P*<0.05). **(D)** Bar plots showing the frequency of overlap between all the TAD groups described in (C) and genome segments within A/B compartment categories described in Figure 2C. **(E-F)** Box plot showing ATAC-seq accessibility gain (**E**) and changes in H3K9me3 ChIP-seq signal (**F**) upon H1 KD (+/-Dox) within the TAD groups described in (B). (***) P < 0.001; (**) P < 0.01; (*) P < 0.05 (Mann-Whitney test). Related to Figure 5.

Supplementary Figure 9. Characterization of TADs presenting a coordinated regulation of gene expression upon H1 KD. (A) TADs with ≥4 genes where at least 80% of genes are down- (left) or up-regulated (right) with FC<-1 or FC>1, respectively, upon H1 KD (total N=294). Log2 of gene expression fold-change (FC) is shown. TADs are ordered from low to high abundance of genes per TAD. Dashed lanes indicate FC=-1.4 or FC=1.4. Red dots represent ISGs. **(B)** TAD groups by proportion of genes per TAD with positive FC upon H1 KD. Only TADs with ≥4 genes were considered. **(C-D)** Box plots showing the expression levels -/+Dox (C) and fold-change (D) within TADs in the 10 groups described in (B). The number of TADs within each group is indicated in (C). (***) P < 0.001 (Wilcoxon signed-rank test). **(E-F)** Box plots showing the GC content (E) and number of genes per TAD (normalized to TAD length and multiplied by the average TADs length) (F) in the 10 TAD groups described in (B). **(G)** Box plots showing the H1X and H1.4 ChIP-seq signal in untreated cells within TADs in the 10 groups described in (B). **(H)** Box plots showing the H1 variants input-subtracted ChIP-seq signal ratio in H1 KD (+Dox) compared to WT (-Dox) cells within TADs in the 10 groups described in (B). Kruskal-Wallis test was applied in all boxplot analysis (D-H) to determine if there were statistically significant differences between the groups. One-sample Wilcoxon signed-rank test was subsequently applied to compare each group of TADs against the median value for each analyzed property.

Supplementary Figure 10. Properties of TADs structurally analysed. Properties of the 7 classes of TADs described in Figure 7 in WT (-Dox) and H1 KD (+Dox) conditions: GC content, expression of genes within TADs, input-subtracted H1.2 and H1.4 ChIP-seq signal, ATAC-seq signal or normalized peaks count; as well as changes +Dox/-Dox within the TAD groups (log2 ratio) on gene expression, H1.4 signal, and ATAC-seq signal. The differences between WT and H1 KD conditions were examined using the Wilcoxon signed-rank test. The global differences between the groups of TADs showing +Dox/-Dox changes were determined by applying a Kruskal-Wallis test. One-sample Wilcoxon signed-rank test was subsequently applied to compare each group of TADs against the median value for each analyzed property. (***) P < 0.001; (**) P < 0.01; (*) P < 0.05.

Supplementary Figure 11. RNA-seq analysis of H1 KD T47D cells. (A) Venn diagram showing overlap between the number of genes down- (left) or up-regulated (right) in H1.4, H1.2 or multiH1 KD (and not changing in random shRNA ±Dox samples) established

by RNA-seq (FC≥±1.4 and *P*<0.05). RNA-seq data has been re-analyzed as described in Supplementary methods for this study. **(B)** Volcano plot of gene expression changes upon H1.4, H1.2 or multiH1 KD measured by RNA-seq. Log2 of fold change is plotted against the minus log10 of *P*-value. Pink dots mark genes with a FC≥±1.4 and *P*<0.05; the number of genes are indicated within the plots. Genes with a FC≥±1.4 and *P*<0.001 are labeled. Red dots mark Interferon stimulated genes (ISGs). The number of genes down- or up-regulated in H1.4, H1.2 or multiH1 KD (FC≥±1.4 and *P*<0.05) that correspond to ISGs are indicated in the table included. ISGs are defined as genes that appear in the Interferome database (version 2.01; Rusinova et al. 2013 Nucl.Ac.Res. 41:D2040-46) in over 5 experiments and that are stimulated by interferon in more than 50% of them with a FC≥2.

Supplementary Figure 12. Coordinated gene expression within TADs upon H1 KD. Fold change +Dox/-Dox (log2) is shown for all coding and non-coding genes present within a representative TAD containing 90-100% of genes with negative (**A**) or positive (**B**) FC, respectively, upon multiH1 KD. Gene expression changes upon H1.2 or H1.4 KD are shown for comparison. Genes are ordered according to their position within the genome. Blue lanes indicate FC=-1.4 or FC=1.4. Red asterisk represents ISGs. Related to Figure 6C.

SUPPLEMENTARY METHODS

RNA extraction and reverse transcriptase (RT)-qPCR

Total RNA was extracted using the High Pure RNA Isolation Kit (Roche). Then, cDNA was generated from 100 ng of RNA using the Superscript First Strand Synthesis System (Invitrogen). Gene products were analyzed by qPCR, using SYBR Green Master Mix (Invitrogen) and specific oligonucleotides in a Roche 480 Light Cycler machine. Each value was corrected by human GAPDH and represented as relative units. Specific qPCR oligonucleotide sequences used as previously described (22).

Total RNA-Seq

Library preparation:

Total RNA-Seq on T47D H1.2sh -/+Dox cells was performed. According to the manufacturer's instructions, the first step involves the removal of ribosomal RNA (rRNA) using target-specific oligos and RNase H reagents to deplete both cytoplasmic (5S rRNA, 5.8S rRNA, 18S rRNA and 28S rRNA) and mitochondrial ribosomal RNA (12S rRNA and 16S rRNA) from total RNA preparations. Following SPRI beads purification, the RNA is fragmented into small pieces using divalent cations under elevated temperature. The cleaved RNA fragments are copied into first strand cDNA using reverse transcriptase and random primers, followed by second strand cDNA synthesis using DNA Polymerase I and RNase H. This process removes the RNA template and synthesizes a replacement strand, incorporating dUTP in place of dTTP to generate ds cDNA. These cDNA fragments then have the addition of a single 'A' base and subsequent ligation of the adapter. After UDG treatment, the incorporation of dUTP quenches the second strand during amplification. The products are enriched with PCR to create the final cDNA library. The libraries were assessed quality and quantity in two methods: check the distribution of the fragments size using the Agilent 2100 bioanalyzer and quantify the library using real-time quantitative PCR (QPCR) (TaqMan Probe). The qualified libraries were sequenced pair end on the BGISEQ-500/ MGISEQ-2000 System (BGI-Shenzhen, China).

RNA-Seq analysis:

RNA-Seq reads were mapped to the human reference genome (GRCh37/hg19) using HISAT2 v2.2.1 with default parameters and specifying strand-specific information (--rna-strandness RF). SAMtools v1.11 was used to sort BAM files and filter for properly paired-end reads (-f 2). Aligned reads were mapped to Ensembl GRCh37.87 gene annotation with TEtranscripts v2.1.4 (--sortByPos --mode multi --stranded reverse). DESeq2 v1.26.0 was used to identify differentially expressed genes between WT and H1.2 KD cells. Gene expression changes were considered significantly different if the absolute value of the log2(FC) was higher than 1.4 and the adjusted p-value was lower than 0.05. RNA-Seq data from T47D H1.2sh cells treated or not with Dox has been deposited in NCBI's Gene Expression Omnibus and is accessible through GEO Series accession number GSE190158.

Histones extraction

For isolation of total histones, cell pellets were resuspended in 1 ml of hypotonic solution [10 mM Tris–HCl (pH 8.0), 1 mM KCl, 1.5 mM MgCl2, 1 mM PMSF, 1 mM DTT] and incubated on ice for 30 min. The nuclei were pelleted at 10000 × g for 10 min at 4ºC. Sulfuric acid (0.2 M) was added to the pellet to extract the histones on ice for 30 min.

The solution was centrifuged at 16000 × g for 10 min at 4ºC. TCA was added to the supernatant in order to precipitate histones. After >1h ice-incubation precipitate was centrifuged (16000xg 10 min at 4ºC). Precipitate was washed with acetone and finally resuspended in water. Protein concentration was determined by Micro BCA protein assay (Thermo Scientific) and immunoblot was performed (see Methods).

H1 Immunoprecipitation (IP) from total histones

Total histones from T47D H1.4sh Untreated or 6-days Dox-treated cells were extracted as previously described. For IP reaction, 60 µg of histones per condition were incubated overnight at 4ºC in RIPA buffer (150mM NaCl, 50mM Tris pH 7.5, 1%NP-40, 0.5% Sodium Deoxycholate, 0.1%SDS, 1mM EDTA) with protease inhibitors, 5µL of anti-H1.4 ChIP-grade antibody (Invitrogen ref. 702876) and 20µL of Protein A magnetic beads. Unbound histones were washed with RIPA buffer. After 3 washing steps, immunocomplexes were directly eluted from magnetic beads using Loading Buffer (5min 95ºC). Immunoblot analysis were performed using 5 µg of input samples (histones) and 1/6 of total eluted IPed material. After anti-H1.4 IP, only histone H1.4 was detected. Importantly, after H1.4 IP in H1.4 KD conditions (+Dox), no pull-down of other H1 variants was observed by immunoblot, meaning that absence of H1.4 does not result in new putative antibody cross-reactions.

Mass-spectrometry analysis of immunoprecipitated histone H1 variants

Mass-spectrometry (MS) was used to further validate H1 antibody specificity. Chromatin extracted from HeLa or T47D cells was used for immunoprecipitation with different histone H1 variant antibodies or unrelated IgG as a control, in triplicate, following the Rapid Immunoprecipitation Mass-spectrometry of Endogenous proteins (RIME) protocol reported elsewhere (Mohammed et al. 2016 Nat. Protoc. 11:316). RIME uses formaldehyde for cross-linking and sonication for fragmentation of chromatin down to ≈200 bp. The resulting material was on-beads digested with trypsin and submitted to mass-spectrometry on a Orbitrap Fussion Lumos spectrometer (Thermo Scientific) to analyze what histone H1 peptides were detected. Peptides corresponding to the IPed H1 variant were highly enriched compared to other H1 variants (see Suppl. Table 2), despite of the fact that some peptides are ambiguous as are conserved between replication-dependent H1 variants (H1.1 to H1.5) and that other variants could be attached to chromatin fragments IPed with a specific antibody.

Immunofluorescence

Cells were fixed with 4% paraformaldehyde (20 min; room temperature), permeabilized with Methanol (10 min room temperature) and blocked with 5% bovine serum albumin. Primary antibody of interest was incubated overnight at 4ºC. Secondary antibodies conjugated to Alexa fluorophores were incubated 1h at room temperature. The following secondary antibodies were used: anti-rabbit IgG (Alexa 488 or 647); anti-mouse IgG (Alexa 555 or 633). Nuclei were stained with DAPI and coverslips mounted using Pro-long glass (Invitrogen). Samples were visualized in Dragonfly 505 multimodal confocal microscope (Andor Technologies, Inc) applying GPU driven deconvolution. Images were taken using 100x Objective and they were analyzed using ImageJ software. For immunofluorescence quantifications, Corrected Total Cell fluorescence (CTCF) was used, according to the following formula: CTCF = Integrated Density – (Area of the

selected Nuclei x Mean fluorescence of background readings). Area of Nuclei was determined by DAPI staining. An equal number of cells per condition (n>30) were used for quantification.

Cell cycle analysis

T47D multiH1sh Untreated or 6-Day-Dox-treated cells ($1x10^6$ total cells per condition) were washed with cold PBS and fixed in EtOH 70% (overnight at -20ºC). EtOH was removed by centrifugation (200 x g 5min) and cell pellet was resuspend in PBS. PBS was removed and 500 µL of staining solution (20µg/ml propidium iodide, 0.2 mg/ml RNase A in PBS-0.5%Triton X-100) was added 30min at room temperature (at dark) before FACS analysis. Samples were analyzed using a Gallios multi-color flow cytometrer instrument (Beckman Coulter, Inc, Fullerton, CA) set up with the 3-lasers 10 colors standard configuration. Excitation was done using a blue (488nm) laser. Forward scatter (FS), side scatter (SS), red (620/30nm) fluorescence emitted by propidium Iodide (PI) was collected. Red fluorescence was projected on a 1024 monoparametrical histogram. Aggregates were excluded gating single cells by their area vs. peak fluorescence signal. DNA analysis (Ploidy analysis) on single fluorescence histograms was done using FlowJo software 10.8.0 (Becton Dikinson).

Supplementary Table 1. Hi-C experimental statistics. Statistics shown separated by replicates (top table) and merged datasets (middle table for valid pairs and bottom table for filtered reads).

Sample	Total reads	Mapped read-end 1	Mapped read-end 2	Mapped reads	% mapped	Valid reads	% valid
Rep1 -Dox	342,288,245	259,075,154	258,107,758	208,117,630	60.8	152,825,989	44.65
Rep2 -Dox	174,269,331	135,986,461	135,602,178	109,694,504	62.95	93,012,379	53.37
Rep3 -Dox	151,794,765	117,907,548	117,082,420	95,250,499	62.75	75,565,442	49.78
Rep1 +Dox	326,519,453	250,463,260	248,485,925	201,425,511	61.69	155,955,437	47.76
Rep2 +Dox	189,256,710	148,401,462	147,801,442	120,043,103	63.43	102,262,949	54.03
Rep3 +Dox	149,483,281	117,243,624	116,902,071	95,143,251	63.65	80,454,962	53.82

Separated replicates

Merged replicates – valid reads

Sample	Total reads	Valid reads	% valid
Rep123 -Dox	668,352,341	321,403,810	48.09
Rep123 +Dox	665,259,444	338,673,348	50.91

Merged replicates – filtered artifacts

Sample	Random breaks	Self-circle	Too close from RES	Over-represented	Dangling-end	Too large	Extra dangling-end	Error	Too short	Duplicated
Rep123 -Dox	3,057,406	399,186	99,467,116	15,284,567	41,896,379	319,233	32,839,003	1,344,033	11,543,359	7,539,158
Rep123 +Dox	2,750,619	394,632	94,255,761	13,603,507	33,351,995	363,675	28,989,911	166,760	11,403,959	6,818,581

Supplementary Table 2. Mass-spectrometry analysis of histone H1 peptides after immunoprecipitation with H1 variant specific antibodies. Chromatin extracted from HeLa or T47D cells was used for immunoprecipitation with the indicated histone H1 variant antibodies in triplicate and the resulting material was submitted to mass-spectrometry (MS) to analyze what histone H1 peptides were detected (see Supplementary methods). Peptides unique to one variant or common to several variants detected by MS are indicated. Tsum and Csum refer to the number of peptides detected for each class in the IP with H1 antibody or with control unrelated IgG, respectively. FC refers to fold-change enrichment in the specific IP compared to control. The IPed H1 variants shows either a high FC or a high number of detected peptides or both (including ambiguous peptides), compatible with variant-specific antibodies. BFDR: Bonferroni false discovery rate.

**HeLa**				
**H1.2 IP**				
**Peptides**	**T sum**	**C sum**	**FC**	**BFDR**
**H1.2 H1.3 H1.4 H1.5**	47	0	156,67	0,11
**H1.1 H1.2 H1.3 H1.4 H1.5**	82	18	4,56	0,18
H1.X	27	7	3,86	0,1
**H1.2**	**105**	**28**	**3,75**	**0,31**
**H1.2 H1.3 H1.4**	238	86	2,77	0,25
**H1.1 H1.2 H1.3 H1.4 H1.T**	105	42	2,5	0,37
H1.5	233	94	2,48	0,45
H1.4	68	31	2,19	0,46
H1.3 H1.4	32	18	1,78	0,69
**H1.4 IP**				
**Peptides**	**T sum**	**C sum**	**FC**	**BFDR**
**H1.2 H1.3 H1.4 H1.5**	61	0	203,33	0
**H1.1 H1.2 H1.3 H1.4 H1.5**	297	18	16,5	0
**H1.4**	**182**	**31**	**5,87**	**0**
H1.X	40	7	5,71	0,32
**H1.2 H1.3 H1.4**	430	86	5	0
**H1.1 H1.2 H1.3 H1.4 H1.T**	180	42	4,29	0,25
**H1.3 H1.4**	63	18	3,5	0,38
H1.5	299	94	3,18	0,4
H1.2	67	28	2,39	0,28
**H1.5 IP**				
**Peptides**	**T sum**	**C sum**	**FC**	**BFDR**
**H1.2 H1.3 H1.4 H1.5**	17	0	56,67	0,12
H1.0	2	0	6,67	0,55
**H1.1 H1.2 H1.3 H1.4 H1.5**	78	18	4,33	0,17
**H1.5**	**271**	**94**	**2,88**	**0,39**
H1.X	19	7	2,71	0,37
H1.2	52	28	1,86	0,47
H1.2 H1.3 H1.4	159	86	1,85	0,72
H1.4	57	31	1,84	0,48
H1.1 H1.2 H1.3 H1.4 H1.T	66	42	1,57	0,72
H1.3 H1.4	25	18	1,39	0,7
**H1.X IP**				
**Peptides**	**T sum**	**C sum**	**FC**	**BFDR**
**H1.X**	**384**	**12**	**32**	**0**
H1.1	10	1	10	0
H1.4	31	23	1,35	0,47
H1.5	110	82	1,34	0,49
H1.2	13	10	1,3	0,45
**T47D**				
**H1.0 IP**				
**Peptides**	**T sum**	**C sum**	**FC**	**BFDR**
**H1.0**	**687**	**0**	**2290**	**0**
H1.3 H1.4	65	0	216,67	0
H1.X	38	0	126,67	0
H1.3	23	0	76,67	0
H1.1 H1.2 H1.3 H1.4 H1.5	20	0	66,67	0
H1.4	36	2	18	0
H1.5	160	11	14,55	0
H1.2 H1.3 H1.4	124	13	9,54	0
H1.2	37	5	7,4	0
H1.1 H1.2 H1.3 H1.4 H1.T	70	10	7	0

Suppl. Figure 1

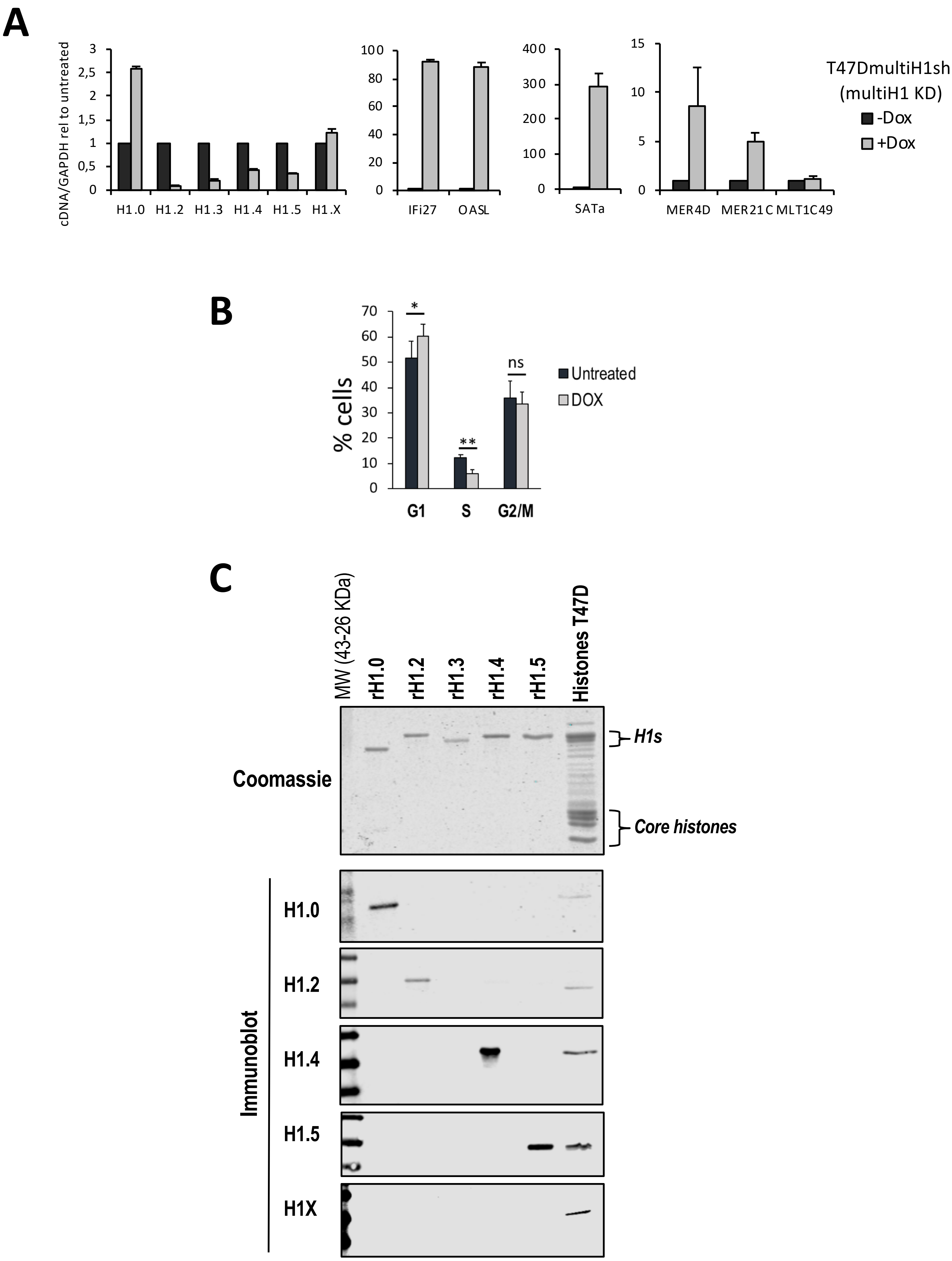

Suppl. Figure 1

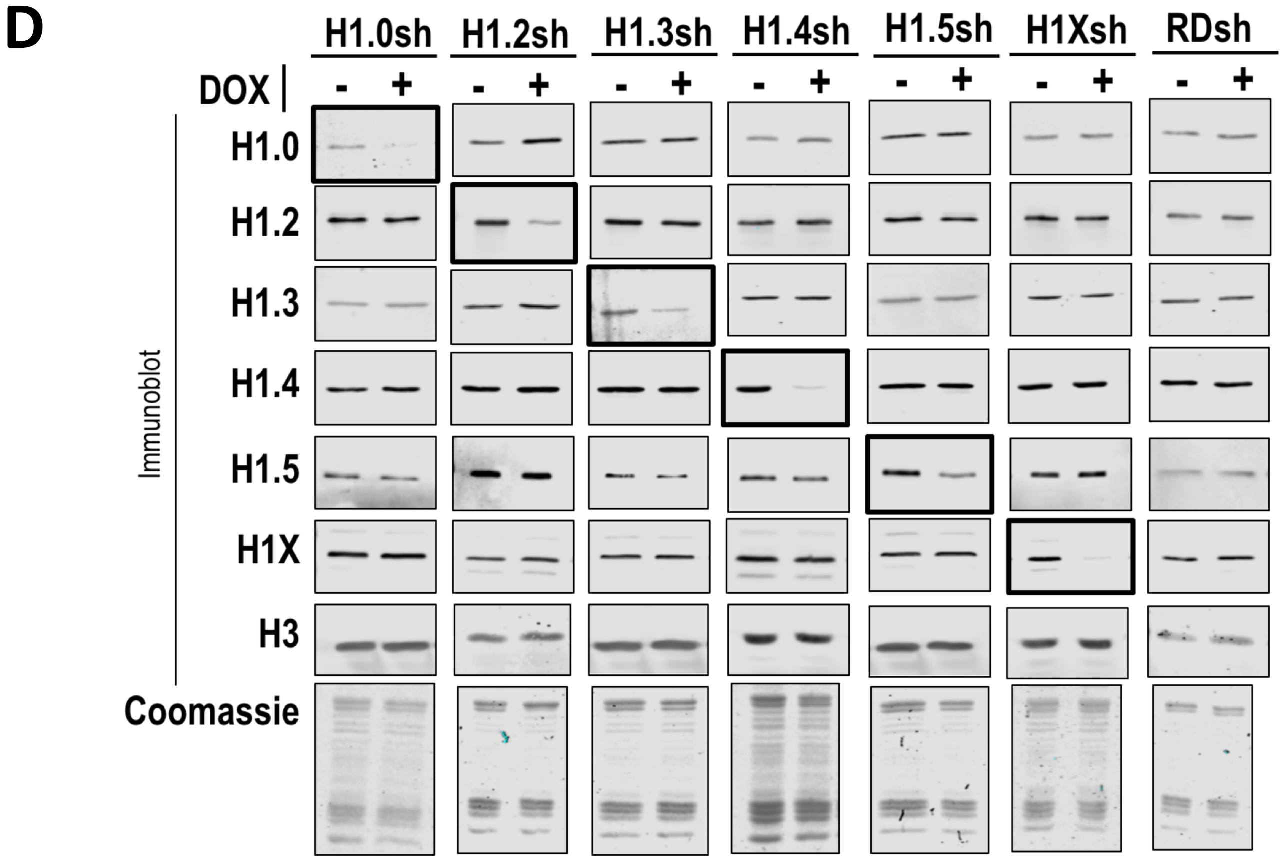

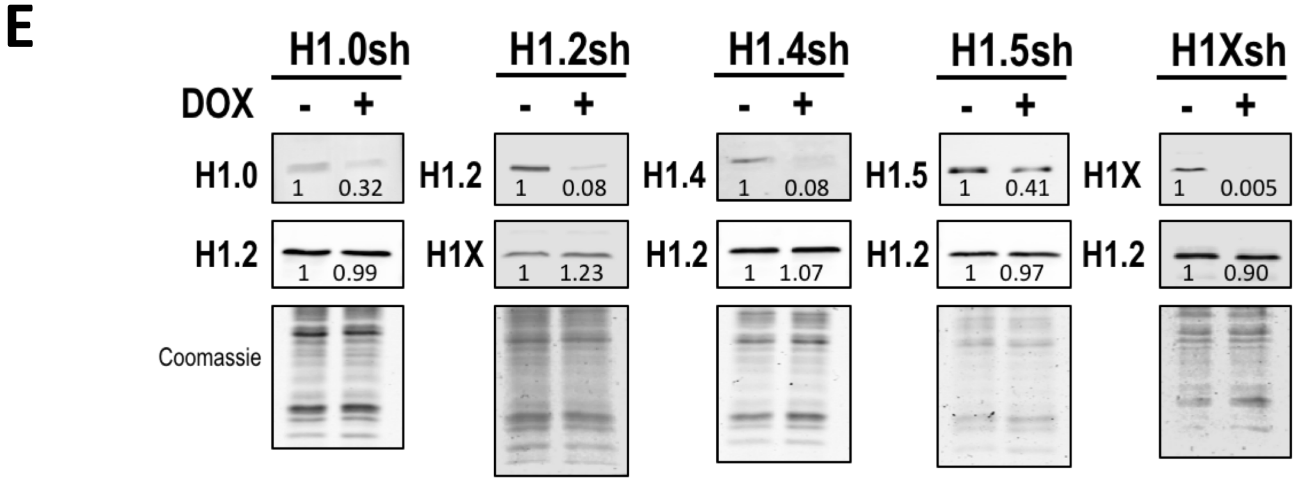

Suppl. Figure 1

F

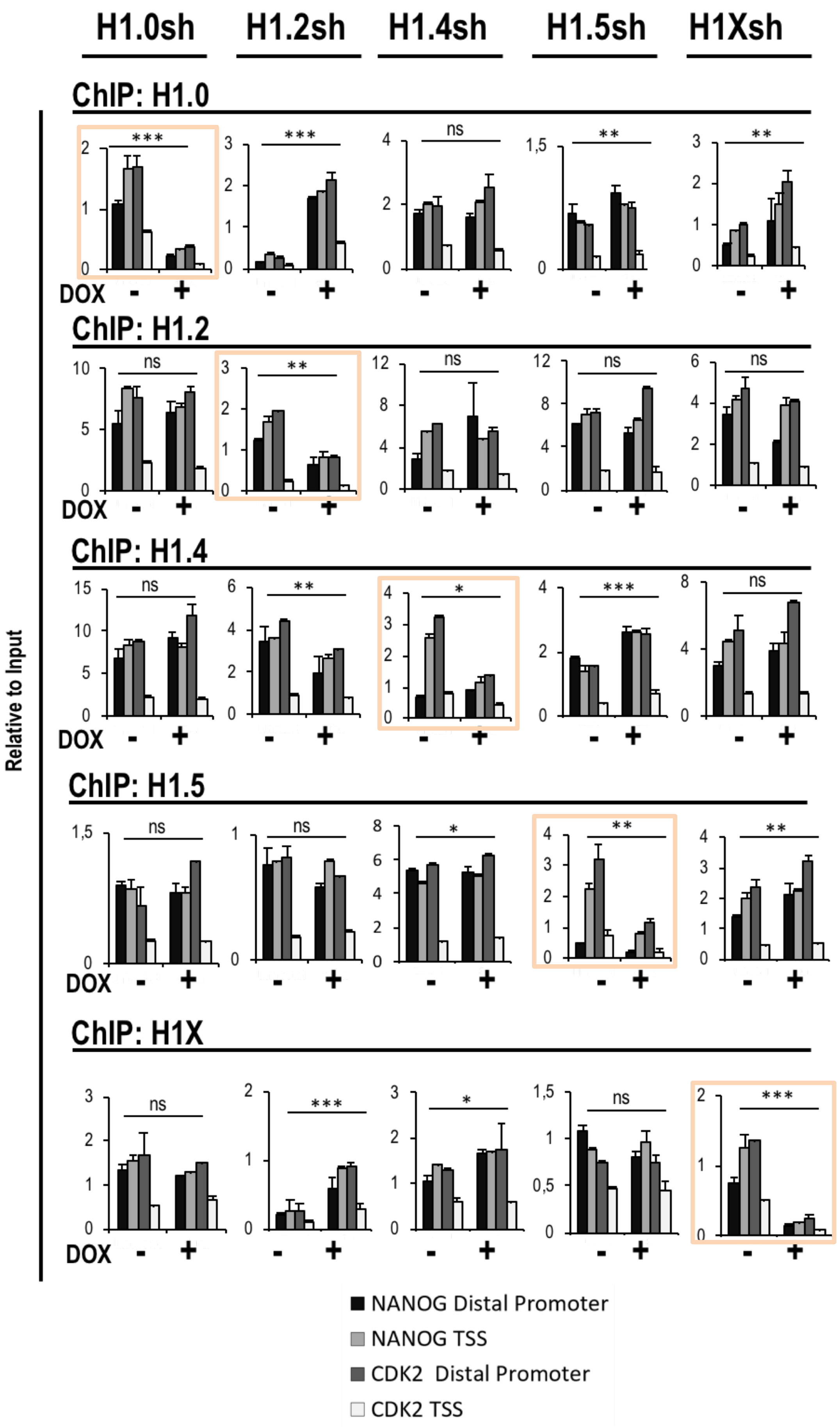

Suppl. Figure 1

G

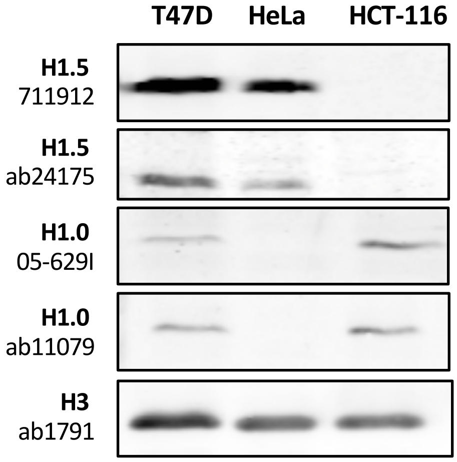

H

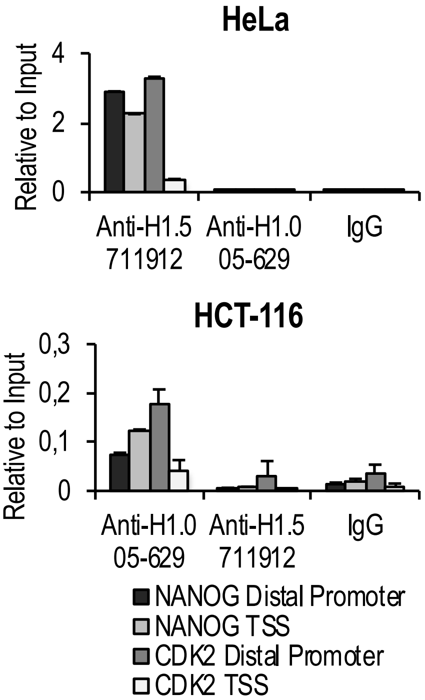

I

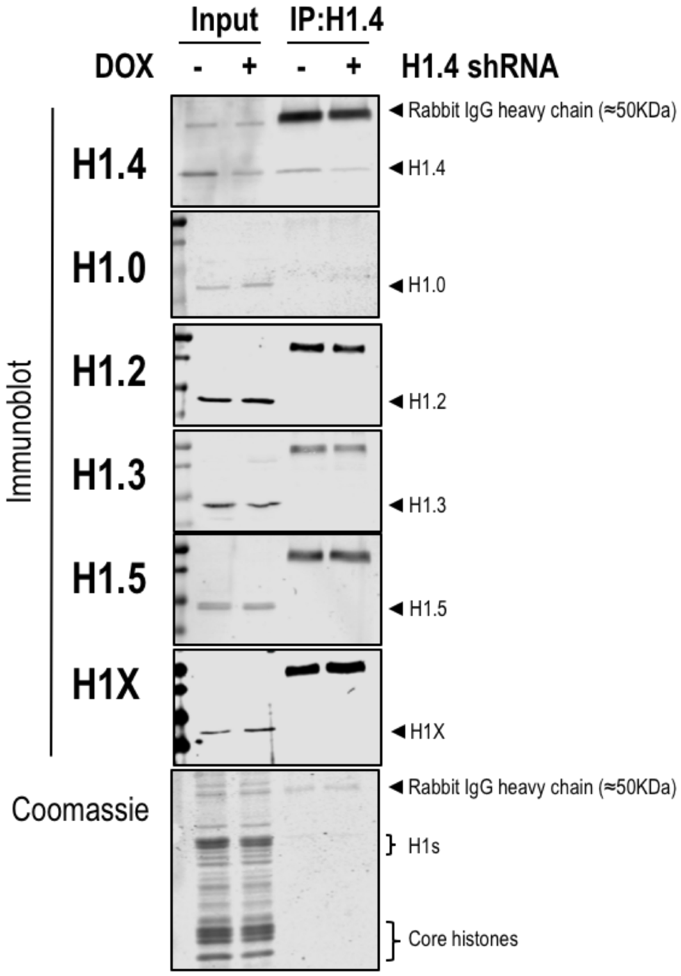

Suppl. Figure 2

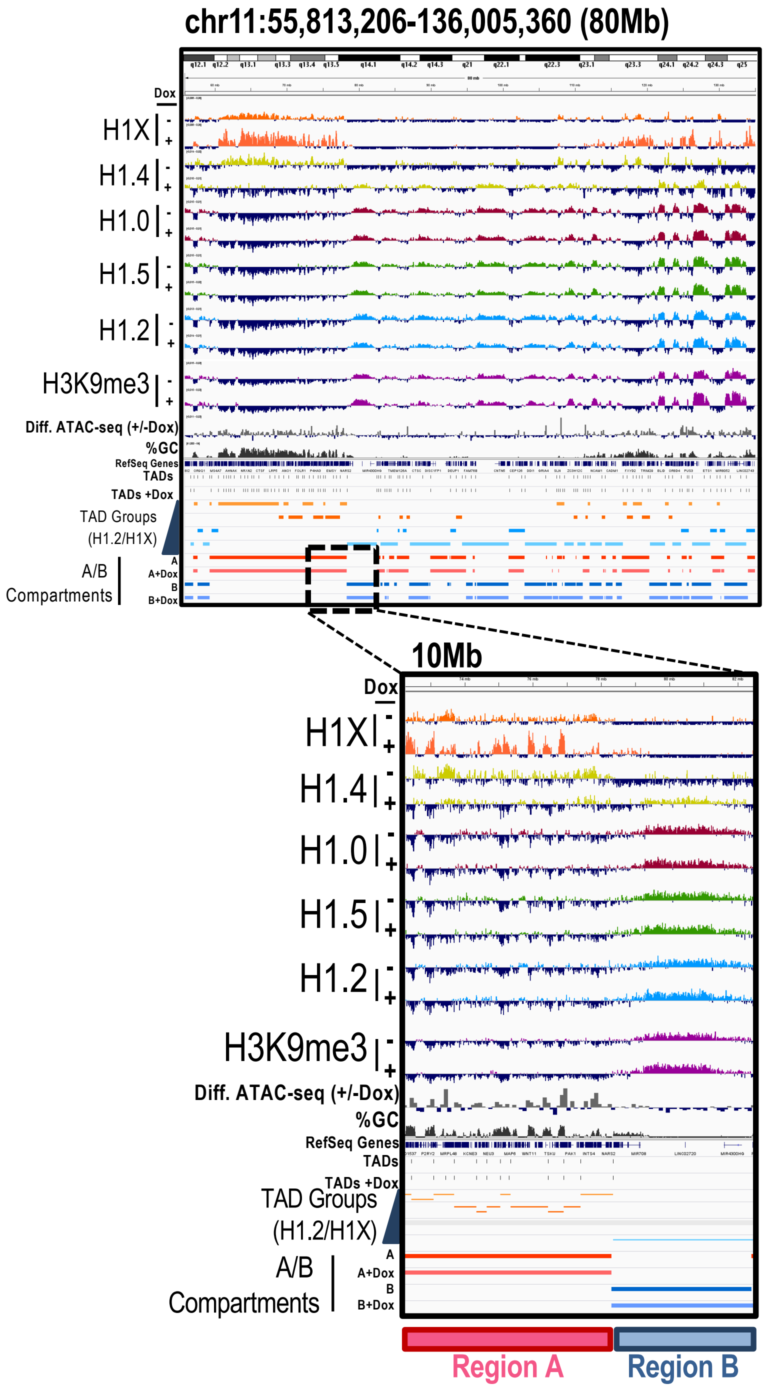

Suppl. Figure 3

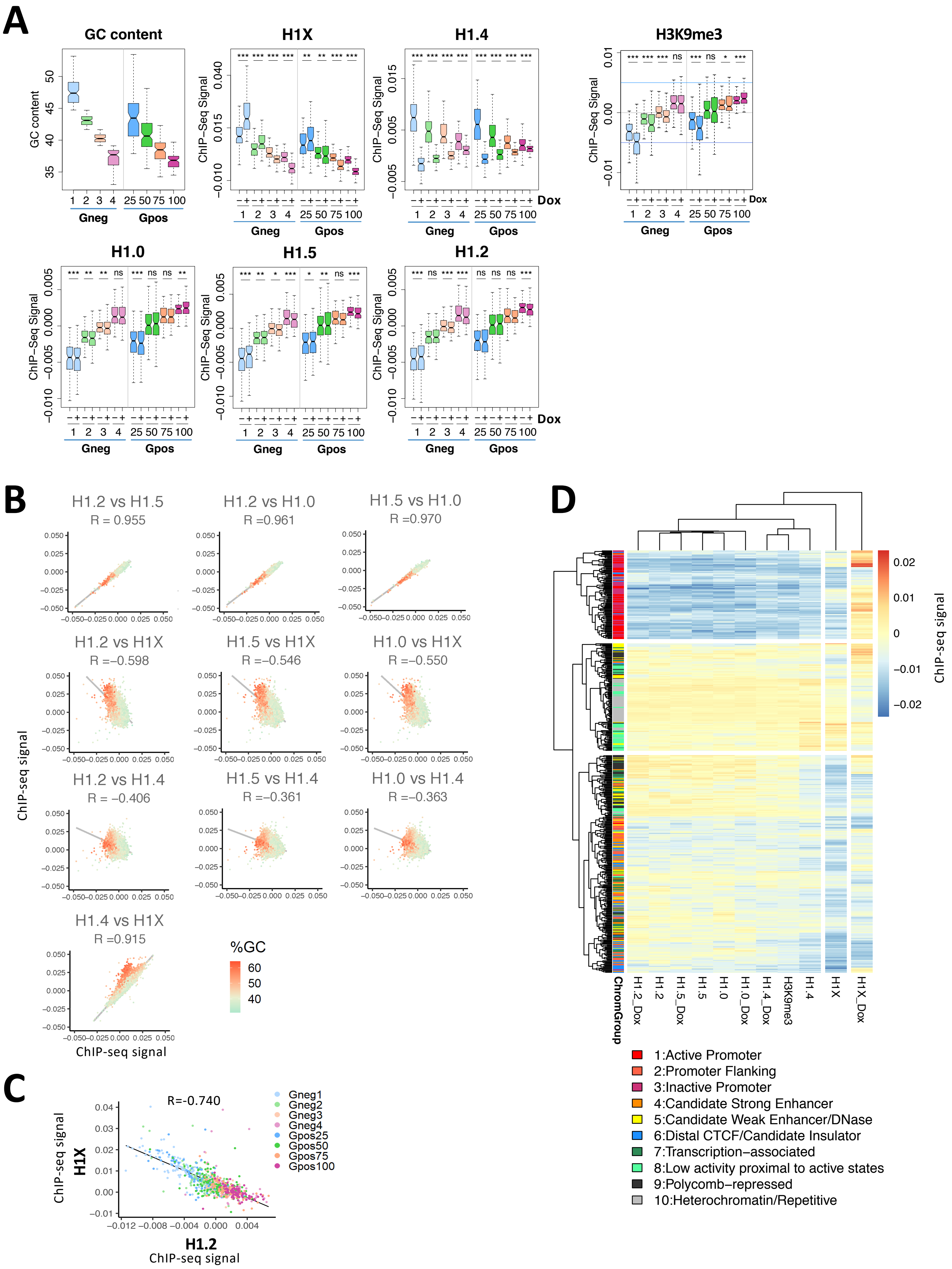

Suppl. Figure 4

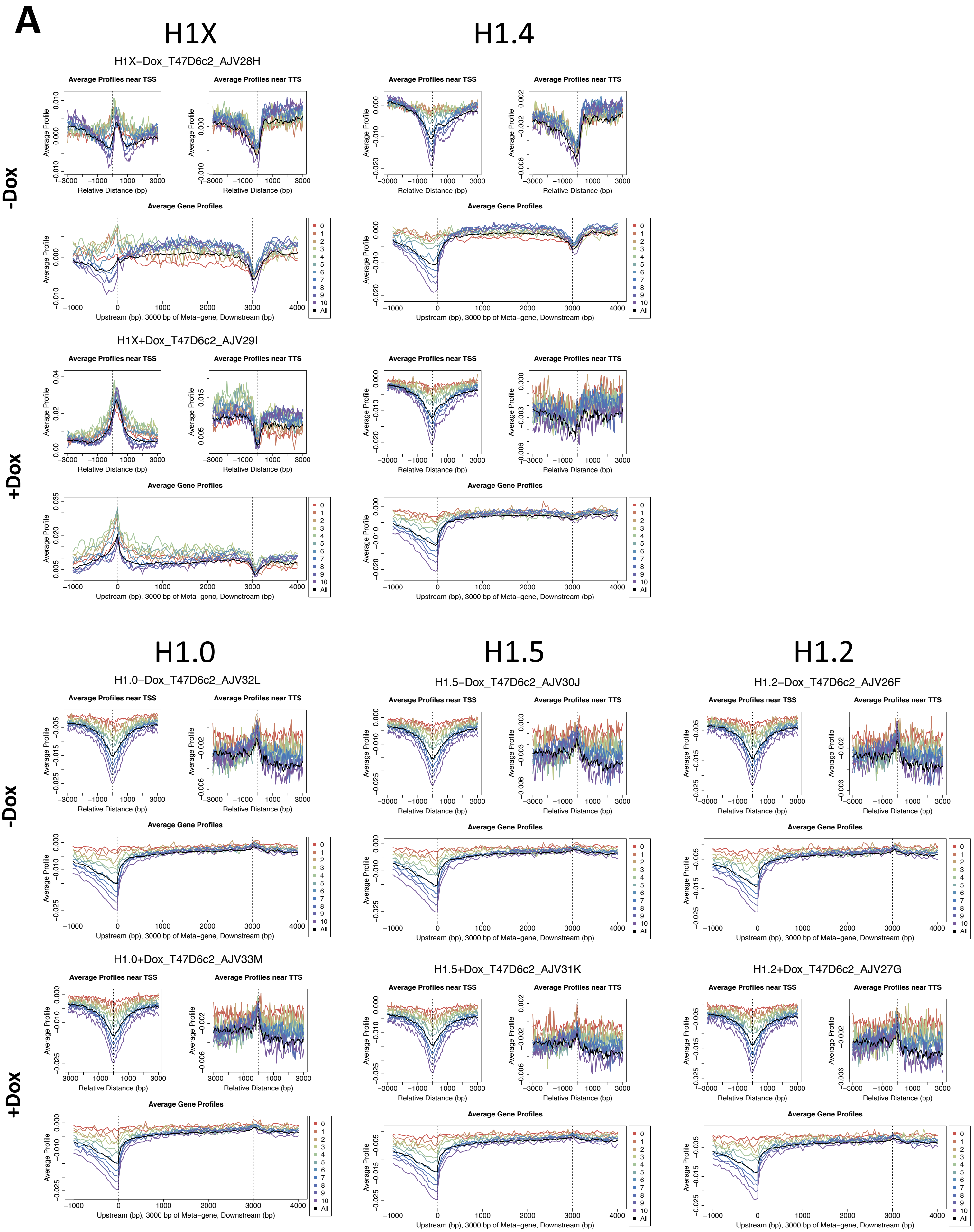

Suppl. Figure 4

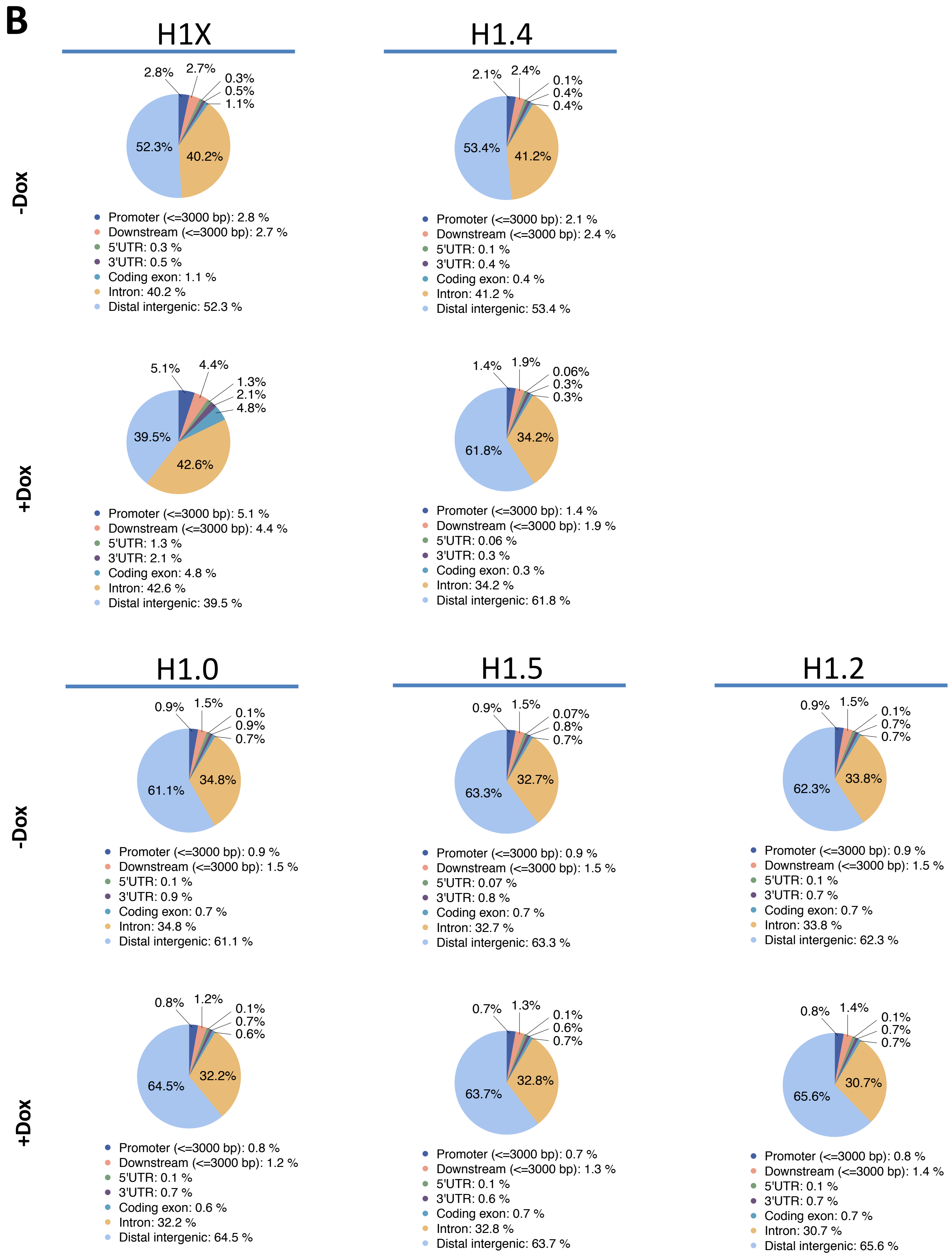

Suppl. Figure 5

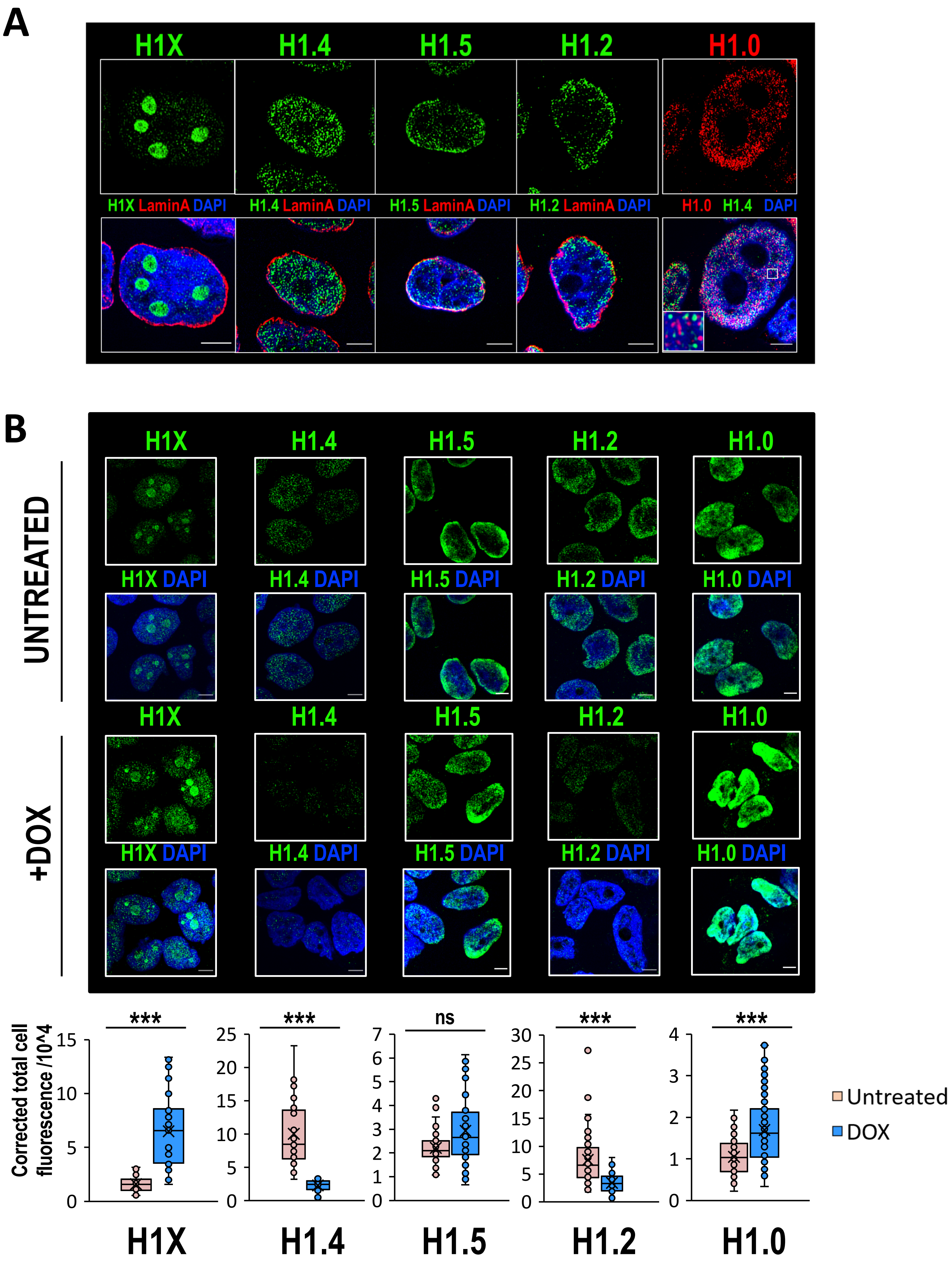

Suppl. Figure 6

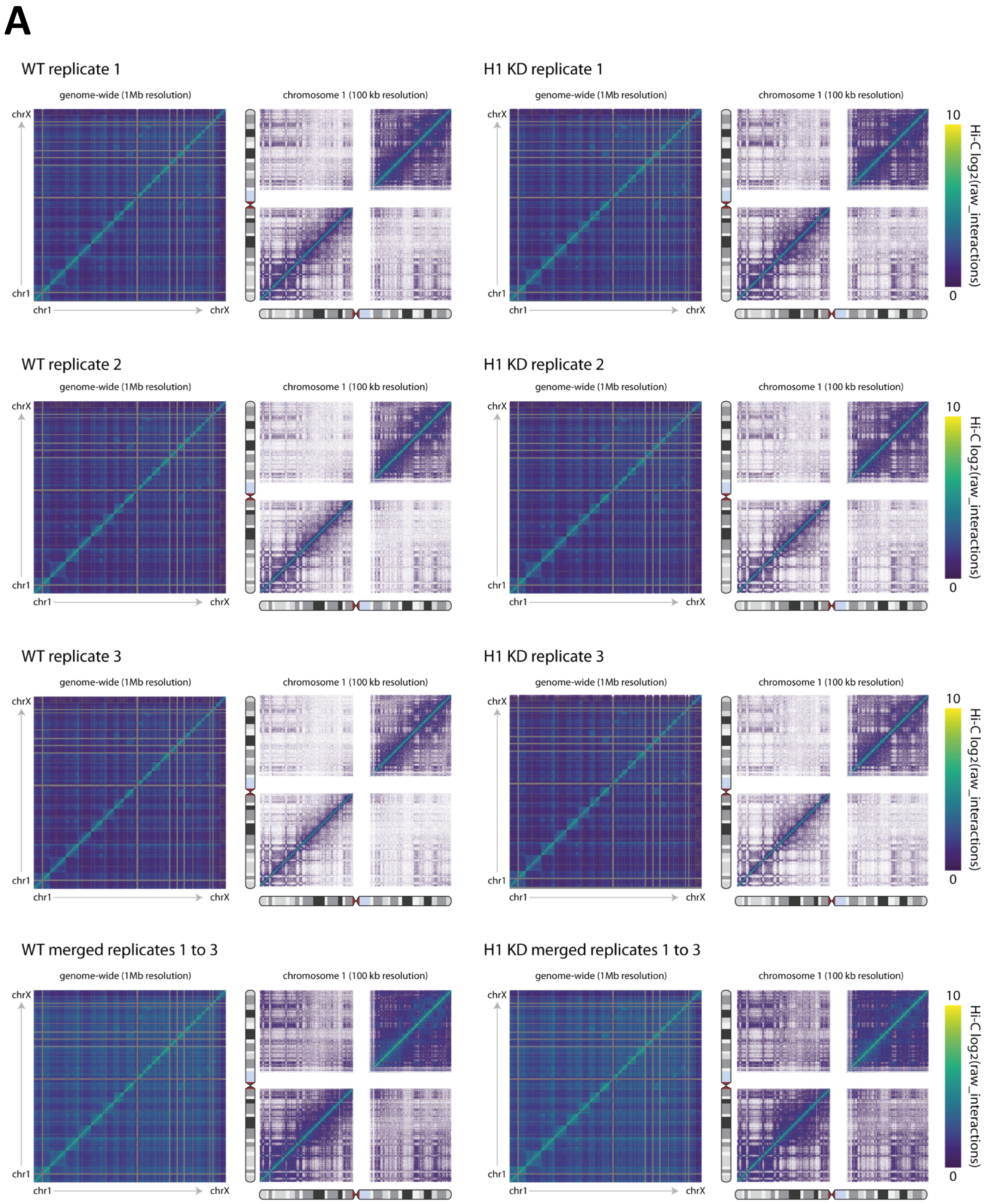

Suppl. Figure 6

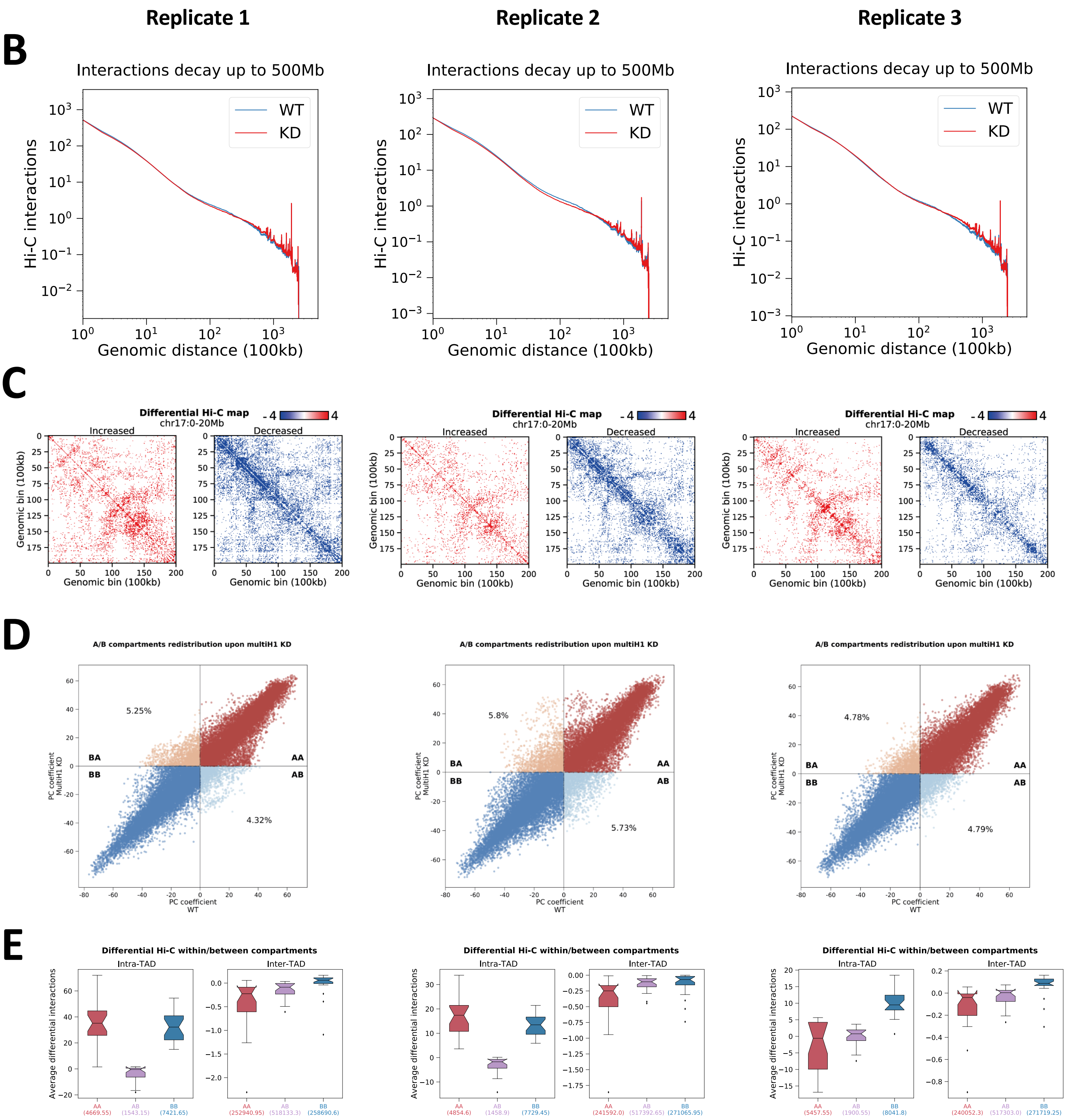

Suppl. Figure 7

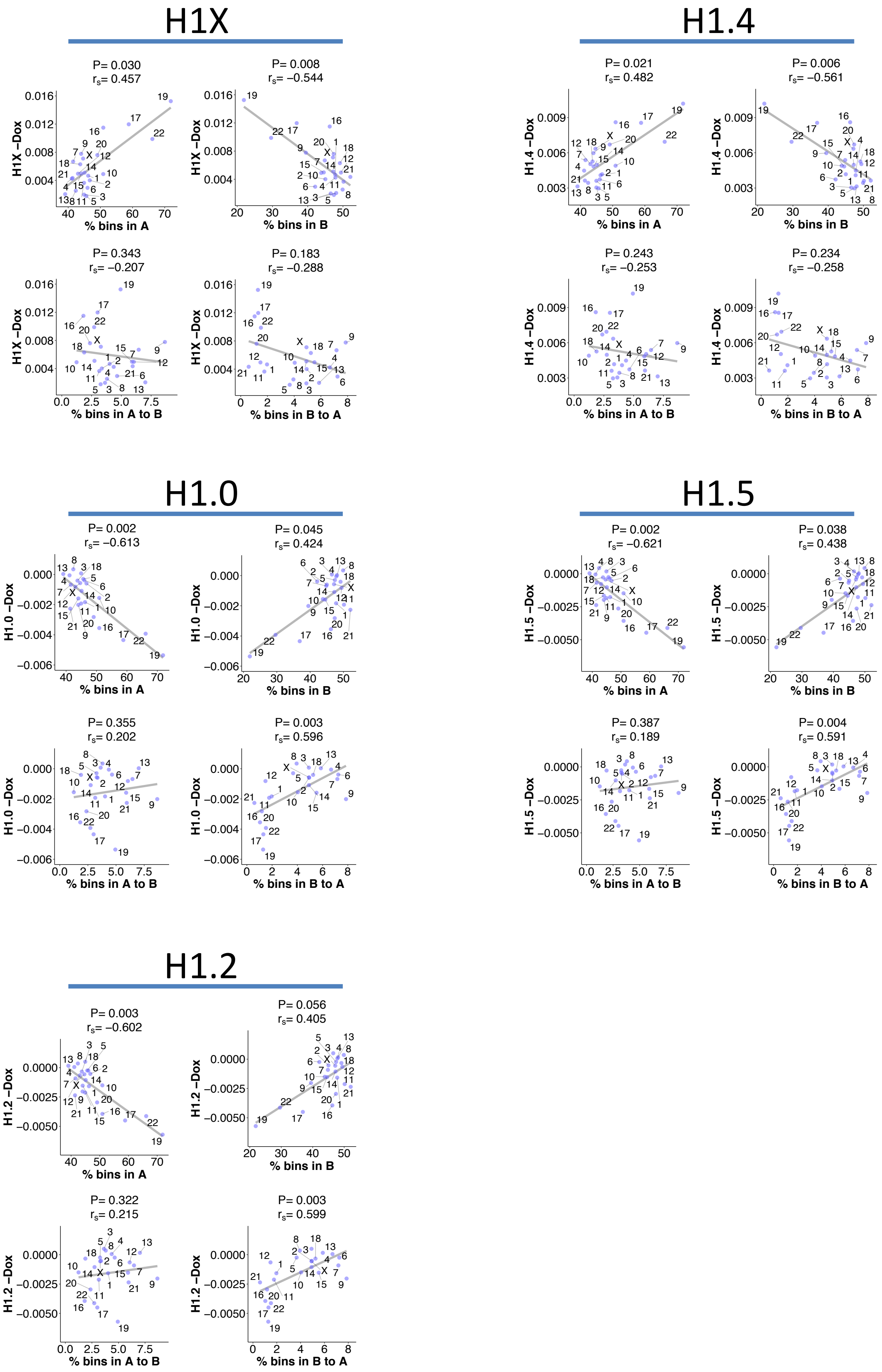

Suppl. Figure 8

A

FC ≥ ±1.4		Upregulated Genes										
		0	1	2	3	4	5	6	7	8	9	
Downregulated Genes	0	1806	429	77	16	6	3	0	0	0	0	2337
	1	382	121	25	8	0	1	0	0	1	0	538
	2	91	23	8	4	1	1	1	0	0	1	130
	3	20	13	1	3	2	1	0	0	0	0	40
	4	14	4	3	2	2	1	0	0	0	0	26
	5	4	4	1	0	1	0	0	1	0	0	11
	6	5	1	0	0	0	0	0	0	0	0	6
	7	1	2	0	0	0	0	0	0	0	0	3
	9	1	0	0	0	0	1	0	0	0	0	2
	10	2	0	1	0	0	0	0	0	0	0	3
	19	0	0	0	0	0	0	1	0	0	0	1
	30	0	0	0	0	0	1	0	0	0	0	1
		2326	597	116	33	12	9	2	1	1	1	3098

Up 531
Dw 520
Up+Dw 241
Subtotal 1,292 (42% of total TADs)
No change 1,806 (incl. 70 no-genes)

B

FC ≥ ±2		Upregulated Genes					
		0	1	2	3	4	
Downregulated Genes	0	2736	158	12	5	1	2912
	1	143	13	2	0	0	158
	2	19	2	1	0	0	22
	3	4	0	0	1	0	5
	18	1	0	0	0	0	1
		2903	173	15	6	1	3098

Up 176
Dw 167
Up+Dw 19
Subtotal 362 (12% of total TADs)
No change 2,736 (incl. 70 no-genes)

C

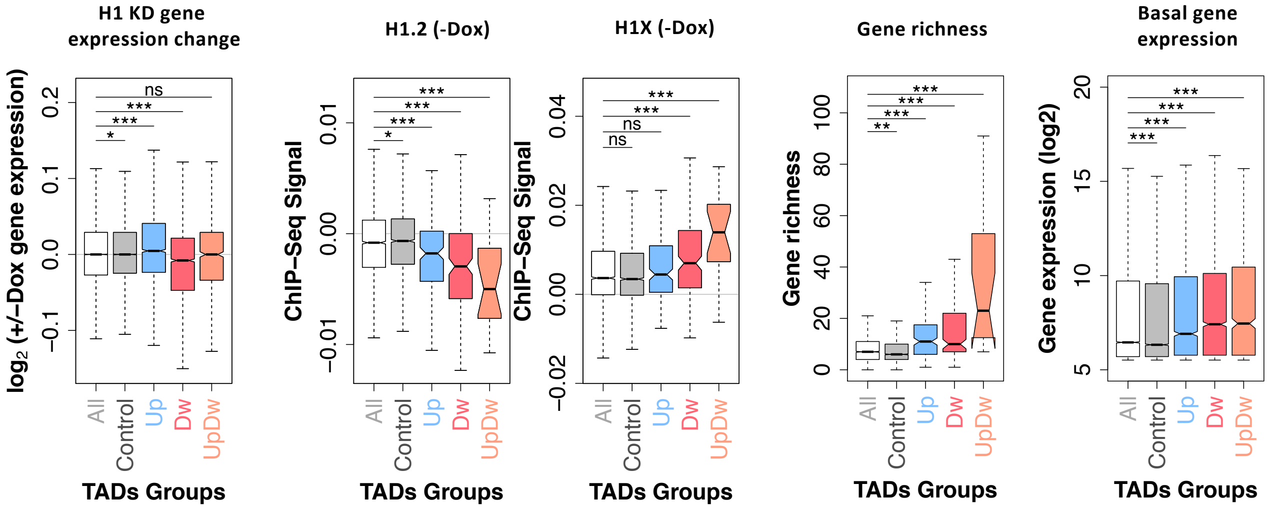

D

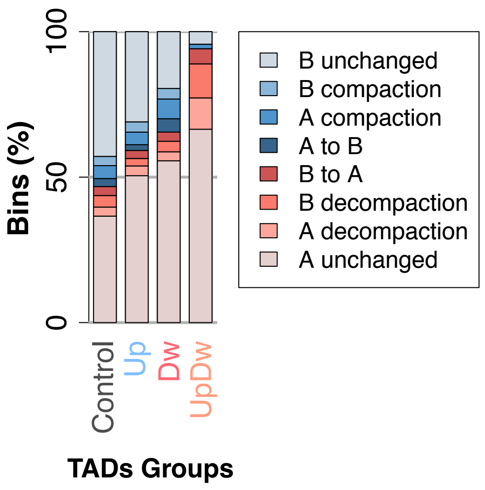

E

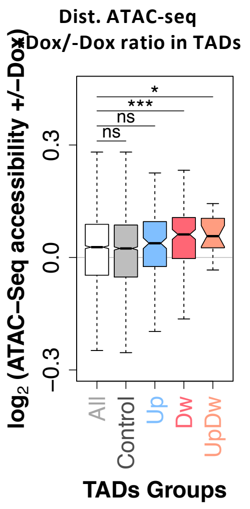

F

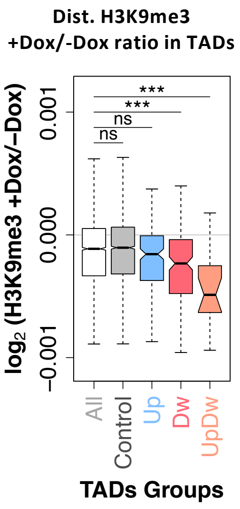

Suppl. Figure 9

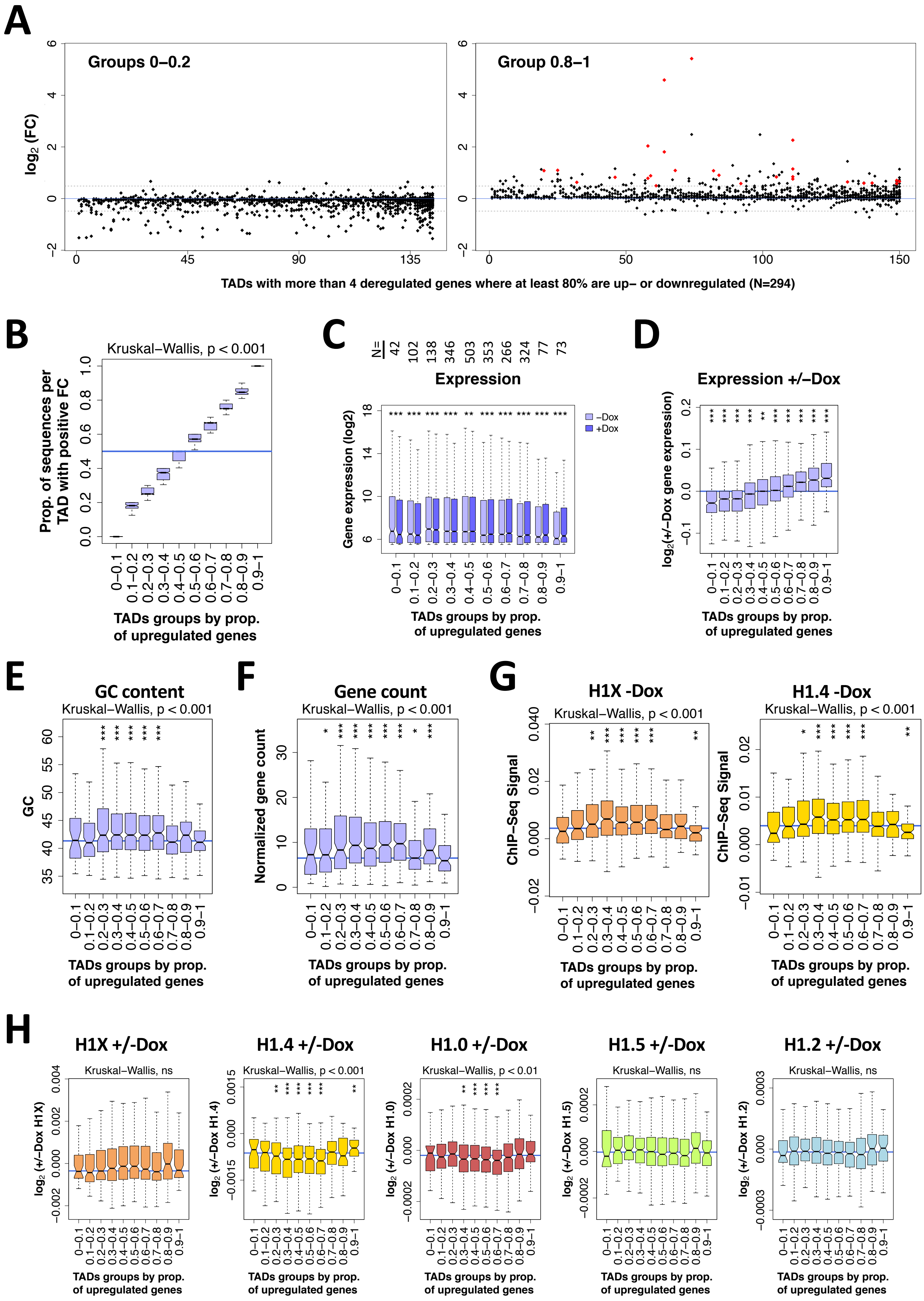

Suppl. Figure 10

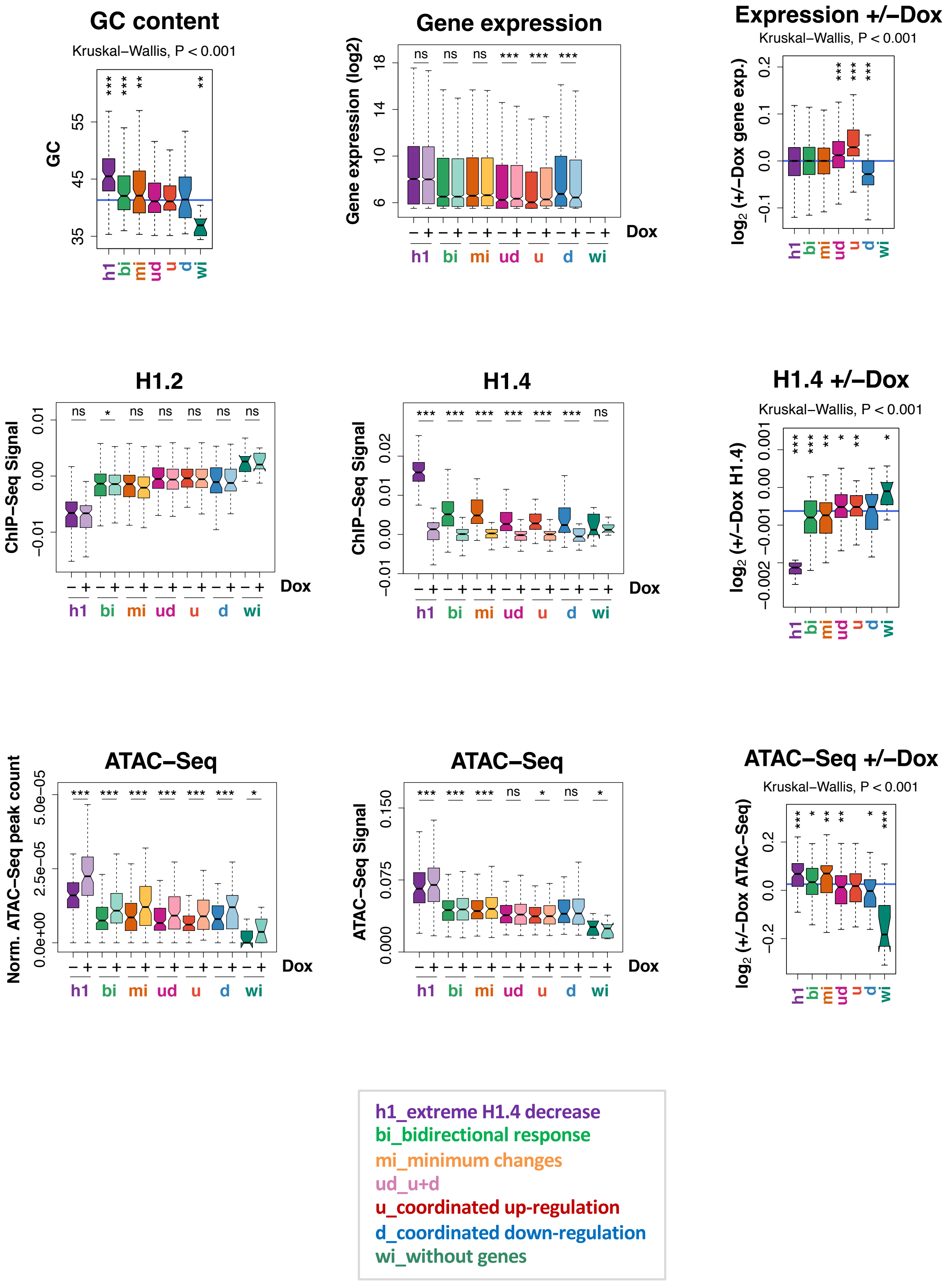

Suppl. Figure 11

A

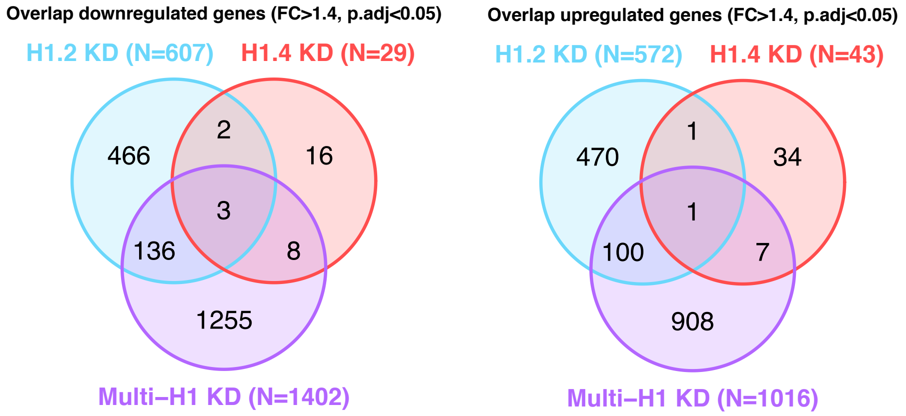

B

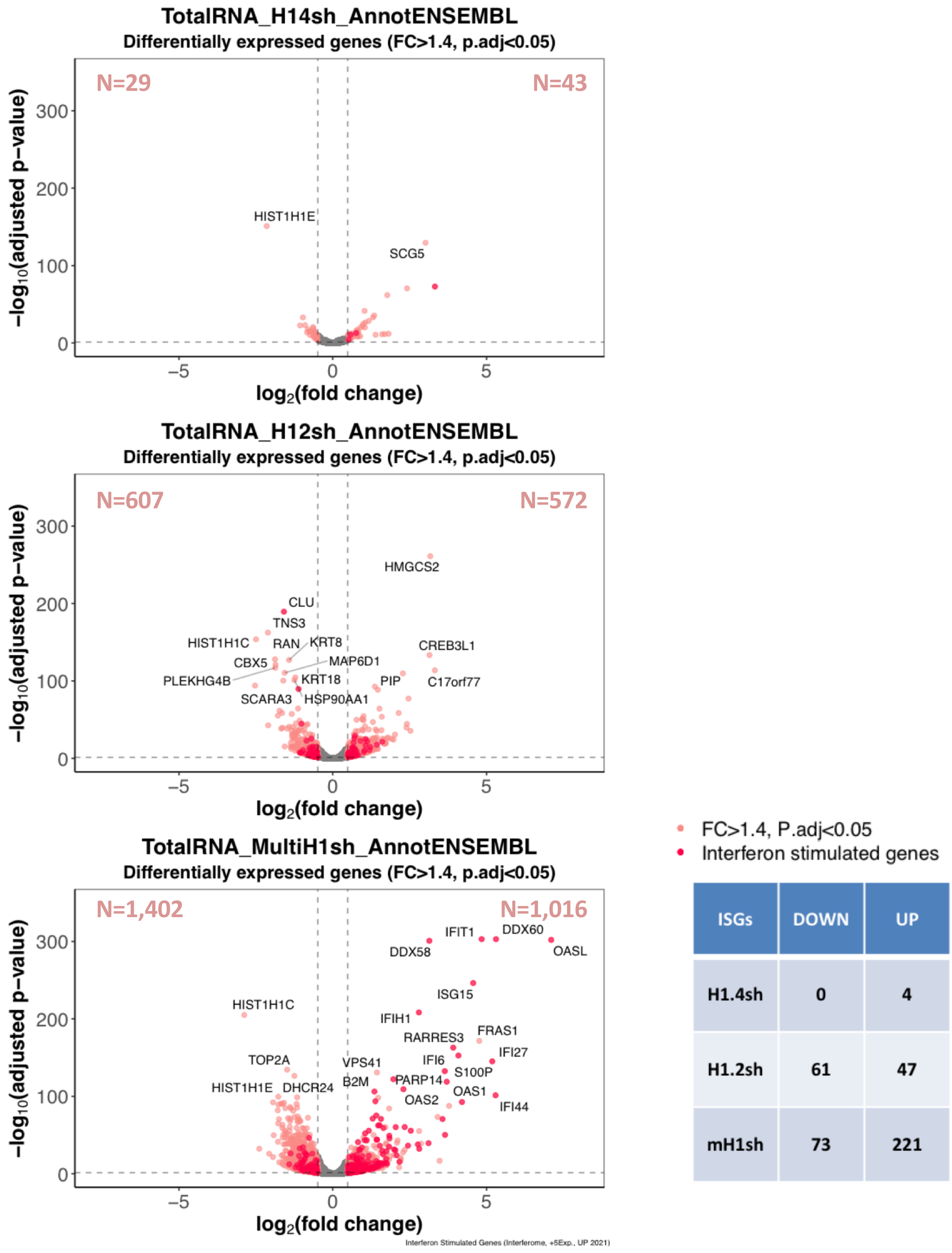

ISGs	DOWN	UP
H1.4sh	0	4
H1.2sh	61	47
mH1sh	73	221

Suppl. Figure 12

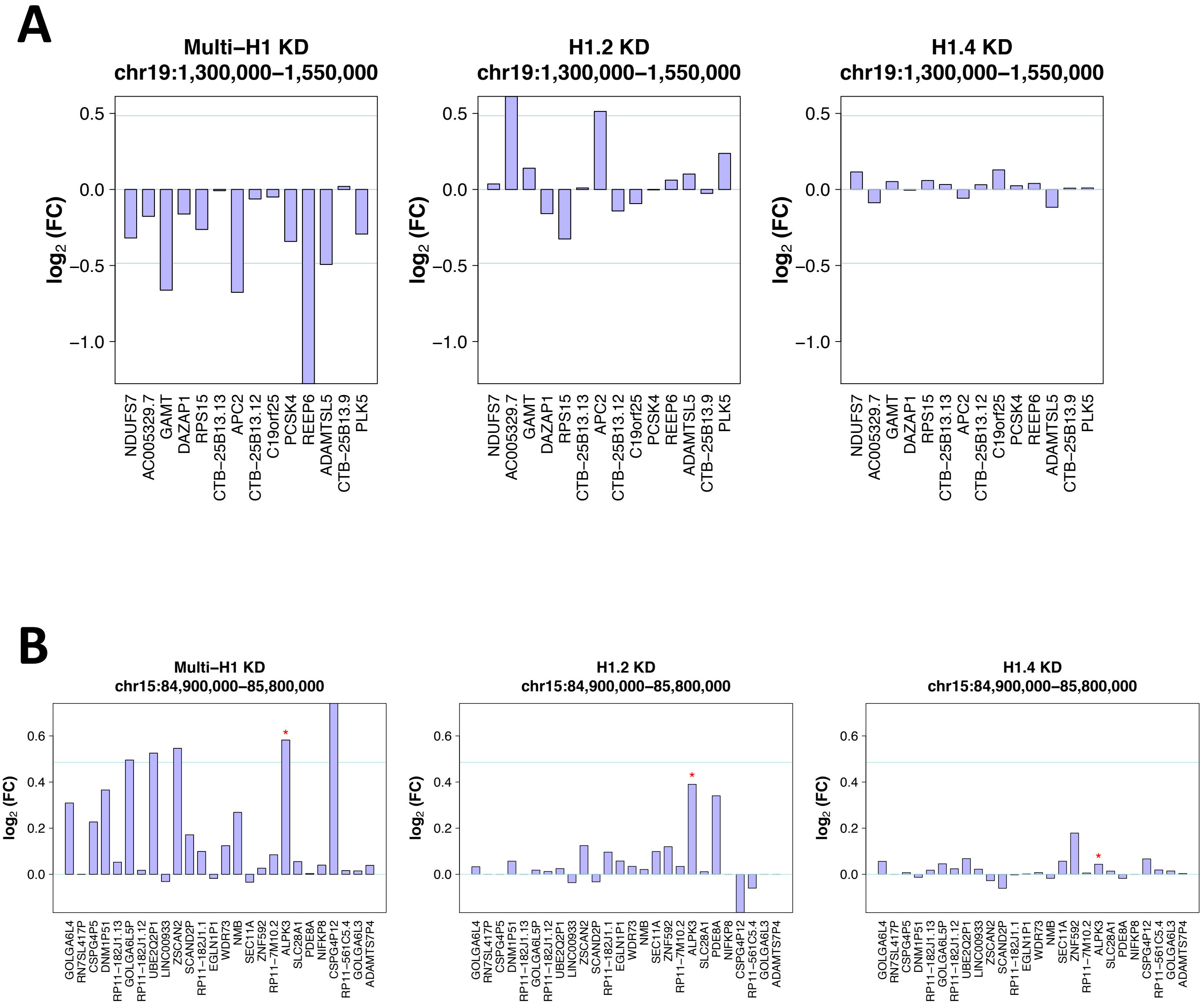

Supplement: gkac226_Supplemental_File [file gkac226_supplemental_file.pdf]
